# Supplementary figures and images for: Mechanical forces stimulate Golgi export
Source: J Cell Biol. 2026 Jul 27;225(10):e202510026. doi: 10.1083/jcb.202510026 (PMC13404088; doi:10.1083/jcb.202510026)

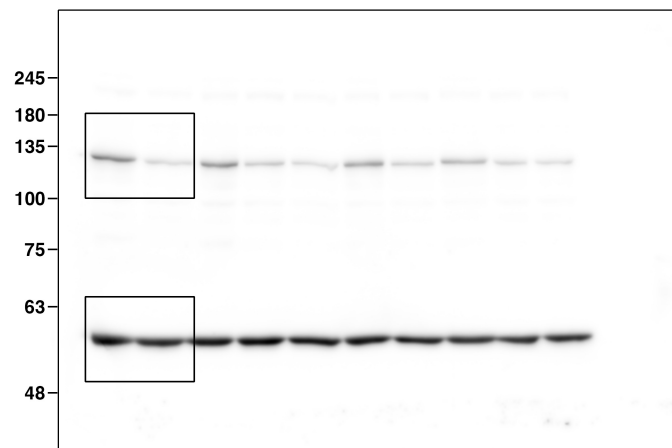

Supplement: SourceData FS1 — is the source file for Fig. S1. [file jcb_202510026_sourcedatafs1.pdf]
