# Supplementary material for: Mechanical forces stimulate Golgi export
Source: J Cell Biol. 2026 Jul 27;225(10):e202510026. doi: 10.1083/jcb.202510026 (PMC13404088; doi:10.1083/jcb.202510026)
Supplement: Review History [file jcb_202510026_review_history.pdf]

JCB

Mechanical forces stimulate Golgi export

Chandini Bhaskar Naidu, Javier Vera Lillo, Eugenia Almacellas, Anabel-Lise Le Roux, Sabine Bardin, Nicolas Mateos, Jessica Angulo-Capel, Adam Wolowczyk, Yugo Terashima, Yuichi Wakana, Pere Roca-Cusachs, Maria Garcia-Parajo, Franck Perez, Bruno Goud, Jean-Baptiste Manneville, Stéphanie Miserey, and Felix Campelo

Corresponding Author(s): Felix Campelo, Universitat Pompeu Fabra; Javier Vera Lillo, Institute of Photonic Sciences; Jean-Baptiste Manneville, Université Paris Cité; and Stéphanie Miserey, Institut Curie

Review Timeline:

Submission Date:	2025-10-04
Editorial Decision:	2025-11-10
Revision Received:	2026-06-13
Editorial Decision:	2026-06-24
Revision Received:	2026-07-01

Monitoring Editor: Johanna Ivaska

Scientific Editor: Andrea Marat

Transaction Report:

(Note: With the exception of the correction of typographical or spelling errors that could be a source of ambiguity, letters and reports are not edited. The original formatting of letters and referee reports may not be reflected in this compilation.)

DOI: https://doi.org/10.1083/jcb.202510026

November 10, 2025

Re: JCB manuscript #202510026

Felix Campelo
Universitat Pompeu Fabra

Dear Dr. Campelo,

Thank you for submitting your manuscript entitled "Mechanical forces stimulate Golgi export". The manuscript was assessed by expert reviewers, whose comments are appended to this letter. We invite you to submit a revision if you can address the reviewers' key concerns, as outlined here.

You will see that the reviewers all appreciate that your study describes a novel link between mechanical forces and post-Golgi carrier formation. They have varying degrees of requested revisions, which we hope you agree will help improve your study.

Reviewers#1 and #3 shared overlapping concerns about the strength of the data in explaining the observed phenomena or in providing a clear mechanistic link. Reviewer#1 proposes different aspects that you could focus on to strengthen the study. While all of these are important, the shared suggestion by Rev#1 and #3 regarding focusing on investigating focal adhesion and their crosstalk with microtubules seems the best to prioritize.

The suggestions regarding the use of micropatterns Rev#3 and testing hypo- and hyper-acetylated microtubule constructs Rev#1 are relevant but may fall beyond the scope of the study, provided you are able to test the effects of integrin activation (Rev#3) and Golgi exocytic activity or MT acetylation inhibition (Rev#1) to help define the Golgi-focal adhesion feedback/crosstalk. The impact of RhoA activity modulation of CARTs formation should also be tested (Rev#1).

While you are revising your manuscript, please also attend to the following editorial points to help expedite the publication of your manuscript. Please direct any editorial questions to the journal office.

GENERAL GUIDELINES:

Text limits: Character count for an Article is < 40,000, not including spaces. Count includes title page, abstract, introduction, results, discussion, and acknowledgments. Count does not include materials and methods, figure legends, references, tables, or supplemental legends.

Figures: Articles may have up to 10 main text figures. Figures must be prepared according to the policies outlined in our Instructions to Authors, under Data Presentation, https://jcb.rupress.org/site/misc/ifora.xhtml. All figures in accepted manuscripts will be screened prior to publication.

IMPORTANT: It is JCB policy that if requested, original data images must be made available. Failure to provide original images upon request will result in unavoidable delays in publication. Please ensure that you have access to all original microscopy and blot data images before submitting your revision.

Supplemental information: There are strict limits on the allowable amount of supplemental data. Articles may have up to 5 supplemental figures. Up to 10 supplemental videos or flash animations are allowed. A summary of all supplemental material should appear at the end of the Materials and methods section.

Please note that JCB now requires authors to submit Source Data used to generate figures containing gels and Western blots with all revised manuscripts. This Source Data consists of fully uncropped and unprocessed images for each gel/blot displayed in the main and supplemental figures. For assays performed using capillary electrophoresis and/or immunoassay-based detection, authors should instead provide the electropherogram graph(s) for each experiment, plotting fluorescence/chemiluminescence intensity vs. molecular weight/size. Please be sure to provide one Source Data file for each figure gels, blots, and/or capillary electrophoresis assays along with your revised manuscript files. File names for Source Data figures should be alphanumeric without any spaces or special characters (i.e., SourceDataF#, where F# refers to the associated main figure number or SourceDataFS# for those associated with Supplementary figures). For traditional gels and blots, the lanes of the gels/blots should be labeled as they are in the associated figure, the place where cropping was applied should be marked (with a box), and molecular weight/size standards should be labeled wherever possible. For capillary electrophoresis assays, each trace in the graph should be color-coded and labeled to indicate which protein, gene, or sample is being measured (please try to avoid red/green combinations to accommodate our color-blind readers).

Source Data files will be made available to reviewers during evaluation of revised manuscripts and, if your paper is eventually published in JCB, the files will be directly linked to specific figures in the published article.

Source Data Figures should be provided as individual PDF files (one file per figure). Authors should endeavor to retain a minimum resolution of 300 dpi or pixels per inch. Please review our instructions for export from Photoshop, Illustrator, and PowerPoint here: https://rupress.org/jcb/pages/submission-guidelines#revised

The typical timeframe for revisions is three to four months. If you anticipate any difficulties in meeting this aforementioned revision time limit, please contact us and we can work with you to find an appropriate time frame for resubmission. Please note that papers are generally considered through only one revision cycle, so any revised manuscript will likely be either accepted or rejected.

When submitting the revision, please include a cover letter addressing the reviewers' comments point by point. Please also highlight all changes in the text of the manuscript.

We hope that the comments below will prove constructive as your work progresses. We would be happy to discuss them further once you've had a chance to consider the points raised in this letter.

Thank you for this interesting contribution to Journal of Cell Biology. You can contact us at the journal office with any questions at cellbio@rockefeller.edu.

Sincerely,

Johanna Ivaska
Monitoring Editor

Andrea L. Marat
Deputy Editor

Journal of Cell Biology

Reviewer #1 (Comments to the Authors (Required)):

The authors explore the potential connection between mechanical forces and Golgi complex function, particularly in the generation of vesicles destined for the plasma membrane. This is an intriguing topic, as the Golgi apparatus remains relatively understudied in the context of mechanobiology. Although previous studies have shown that the Golgi is a mechanosensitive organelle that responds to factors such as matrix stiffness and cell adhesion, a direct link between mechanical forces and post-Golgi carrier formation has not yet been established. This work therefore addresses an important and timely question, how the Golgi apparatus functions as a mechanosensor that coordinates exocytic trafficking with focal adhesion (FA) assembly and cell adhesion, revealing potential positive feedback mechanisms within this process.
The experiments are well designed and carefully executed, with clear data presentation and appropriate statistical analyses. However, while the authors clearly demonstrate that Golgi carrier biogenesis is linked to mechanical forces, the manuscript currently lacks a clear mechanistic link connecting the observed phenomena. Additional work is needed to establish how the identified processes are causally related.

General comments:
The DAG reporter used is based on the C1 domain of PKD. However, this construct may compete with endogenous PKD for DAG binding, potentially influencing CARTS formation. Moreover, given the feedback loop between active PKD and DAG generation (as shown by Fugmann et al. 2007), the reporter may obscure the true dynamics of DAG levels. As an alternative, the authors can employ alternative reporters that directly monitor PKD activity at the Golgi (e.g. using a genetically encoded PKD reporter), as this would more accurately reflect DAG-related generation of CARTS.
The molecular mechanism linking microtubule (MT) acetylation to DAG levels at the TGN remains insufficiently explored. The authors propose involvement of the GEF-H1/Rho pathway, and indeed, a recent preprint suggests a connection between this pathway and MT acetylation (Roy et al 2024, https://doi.org/10.1101/2024.12.01.626286). Testing the effects of Rho inhibition/activation on CARTS formation and Golgi-dependent exocytic activity would significantly strengthen this hypothesis. Alternatively, employing Rho or GEF-H1 activity reporters could help determine whether Golgi-mediated exocytic activity provides feedback to this signaling pathway.
In addition to pharmacological inhibition with tubacin, employing hypo- and hyper-acetylated microtubule (MT) constructs (Joo et al., 2014) could offer more direct evidence connecting MT acetylation to Golgi-dependent exocytic function, particularly given that HDAC6 acts on multiple substrates beyond tubulin.
Finally, it would be important to assess the impact on focal adhesion dynamics (in addition to cell adhesion) when Golgi exocytic activity or MT acetylation is inhibited. This could help define the feedback relationship between Golgi mechanosensing, vesicle trafficking, and adhesion assembly.

Specific comments:
Figure 3B: The observed drop in Halo-Flipper lifetime at 3 hours post-seeding is unexpected, especially since the lifetime increases again at 4 hours. The authors should provide an explanation for this transient decrease, as it seems inconsistent with the overall trend.
Figure 4B: Please include the p-values for the PLL group to allow proper statistical evaluation and comparison across conditions.
Figure 6A and B: The reported decline in DAG levels measured with the Golgi sensor does not appear to correlate with the observed increase in CARTS formation shown in Figure 1. The authors propose that this may result from DAG incorporation into nascent transport carriers or conversion into other lipids required for membrane fission. However, if DAG is being depleted, it is unclear how CARTS formation remains robust (Fig. 1C). Similarly, treatment with tubacin enhances CARTS formation without affecting DAG levels. These discrepancies suggest that DAG quantification through the current sensor might not fully capture functionally relevant DAG dynamics. As previously mentioned, employing a PKD activity reporter could provide a more direct and reliable measure of CARTS formation, given that PKD activation is tightly coupled to this process.
Figure 7: It would be highly interesting to test whether PKD inhibition, particularly under conditions where cells are plated on fibronectin, affects microtubule (MT) acetylation. Such an experiment could provide valuable insight into the feedback relationship between CARTS formation and MT acetylation discussed earlier.

Reviewer #2 (Comments to the Authors (Required)):

In this paper, the authors show the novel finding that the Golgi apparatus functions as a mechanoresponsive organelle whose secretory output is directly modulated by extracellular mechanical forces. This work establishes a mechanotransduction axis linking the extracellular matrix to directed Golgi-to-plasma membrane (PM) trafficking.
The study demonstrates that mechanical input-specifically increased substrate stiffness, robust cell spreading, or applied equibiaxial strain-significantly enhances Golgi transport carrier biogenesis, notably increasing the exocytosis of CARTS (carriers of the TGN to the cell surface). This augmented Golgi export is essential for cell adaptation, as its inhibition (via Golgicide A or PKD inhibition) or the depletion of the CARTS adaptor TGN46 severely impairs sustained cell spreading and mechanoadaptation.
The paper elucidates a specific molecular and biophysical cascade transducing the mechanical signal:
-Membrane Mechanics. Extracellular forces correlate with a progressive increase in Golgi membrane tension.
-Cytoskeletal Link. Robust spreading enhances microtubule (MT) acetylation, a modification that is sufficient to promote CARTS biogenesis independently of external force when experimentally mimicked (e.g., using tubacin).
-Signaling Axis. MT acetylation feeds into the RhoA/PLCε/DAG/PKD axis, leading to the accumulation of diacylglycerol (DAG) at the Golgi. DAG-mediated activation of Protein Kinase D (PKD) is identified as necessary for the subsequent carrier formation and membrane remodeling.
The authors propose a comprehensive model where forces transduced from focal adhesion (FA) establishment promote MT acetylation, which then activates the RhoA/PLCε/DAG/PKD pathway to boost Golgi export, thus enabling cellular adaptation to its mechanical environment.
Response to Minor Revisions
The following points represent necessary clarifications and data presentation checks required to finalize the manuscript.
-It is noted that while spinning-disk microscopy was conducted at 37 the temperatures for Fluorescence Lifetime Imaging Microscopy (FLIM) and Live-cell TIRF measurements are not explicitly stated.
-Please clearly specify the temperature used for all FLIM and Live-cell TIRF experiments.
-Given that the study focuses heavily on membrane mechanics and lipid dynamics (DAG levels and Golgi membrane tension via Halo-Flipper), it is critical to confirm the temperature. Lipid behavior, including membrane order and phase separation, is highly sensitive to temperature changes, and failure to control or report the temperature, especially between room temperature 37 could introduce confounding variables or make lifetime data incomparable.
-Please ensure that the total number of independent experiments N and the total number of cells or measurements n are clearly provided for the following figures: Figure 1H, Figure 3B/D, Figure 5F, Figure 7F, Figures S2B, and S3B.
-Please clarify precisely what each individual point and/or colored data group on the scatter plots and bar graphs represents (e.g., is each point the median value derived from one cell, or an aggregate measurement from a population of cells, or a biological replicate. This detail is crucial for interpreting the statistical methodology.

Reviewer #3 (Comments to the Authors (Required)):

This work builds on publications describing exocytosis occurs near FAs occurs at exocytic hotspots (a term coined by Yau et al MBoC 2011) spatially controlled by microtubule targeting (first reported in Stehbens et al NCB 2014 identifying Rab6 exocytic hotspots at FA) which coordinates post Golgi secretion at these PM sites (Fourriere et al., 2019).

Here they explore the idea of mechanochemical feedback loops between adhesion, membrane tension (focusing on Golgi but PM would also be implicated) with Golgi-derived biogenesis and exocytosis via mechanical stimuli. While we know a lot about focal adhesions in sensing mechanical signals, there is less known about organelles like the Golgi. (although there has been a

significant shift in our knowledge of nuclear mechanosensing too- I would suggest adding a sentence referring to this near line 6-9 where organelle mechanosensing is first introduced).

I wholeheartedly agree- there is likely a mechanochemical feedback loop linking microtubules, and membrane tension with targeted exocytosis at focal adhesions which enables cells to modify their membrane tension. Golgi membrane mechano-sensitivity will be key to understanding this biology. However, while interesting, the data as presented fails to convincingly connect the phenomena and is largely speculative. They often equate results with biological phenomena that they do not directly test. Several of their experimental manipulations alter CARTS -but do not affect the membrane. I strongly suggest they focus on focal adhesions in this context as they might be the link they are missing in their data. I did enjoy the discussion where they expand on their ideas about membrane exo-endo cytosis balance and their identification of some tool shortfalls- particularly the issue of Flipper probes in Golgi tension measurements. I outline several queries below in more detail which I think would help the authors to better articulate their work in a more comprehensive way. I hope that the authors find this helpful.

CARTS exocytosis at adhesions.
They first image CARTS exocytosis at adhesions via TIRF. They state that the PAUF positive carriers (to mark CARTS) exit the Golgi and dock at FA. To better support this conclusion, I would encourage the authors to quantitate this data- it is live imaging, it should be relatively straight forward to measure proximity relative to FA over time, as well as dock, dwell and fusion measurements. (Although I did see the authors chose to only image focal adhesions once over the 45 min duration).

They then compare CARTS # in cells seeded on PLL vs FN coated substrates. They compare membrane area to CARTS # on the two substrates, concluding that FN attached cells have more CARTS and an increase in area. Q: Is the total surface area of the cell smaller overall? i.e. are the PLL cells not spread, but are higher? A z stack should be able to demonstrate this.

The role of integrin activation
The authors conclude/infer that integrin activation and focal adhesion formation act to coregulate Golgi export- however, do not test any effects on focal adhesion formation nor integrin activity.

They look at focal adhesion formation on 2 vs 30 kPa gels (vinculin) and conclude that as focal adhesion numbers are less, the decrease in CARTS is due to modification in integrin activation (but do not test integrin activity directly). Cell morphology is not always an indicator of integrin activation - see this work where stem cells on soft substrates had higher integrin B1 activation. This requires specific antibodies which should be able to detect the total and activated to support their statement
https://www.pnas.org/doi/10.1073/pnas.1106467108
If they add +Mn or cRGD to modify integrin activation, does this alter CARTs biogenesis?

CARTS and mechanical signals
The authors propose that CARTS export might be mechanically linked. They use tuned substrates and strain. The results are often variable, which suggests maybe they need to consider additional control of the mechanical signal to rule out confounding effects. Here, I think micropatterning would be very effective. Can you tune CARTS export and number by plating cells on micropatterned substrates of various sizes? This could be combined with PAGs and adhesion substrates to control membrane tension associated with spreading. See https://doi.org/10.1242/jcs.075150

Confounding data lacking significance
They compare CARTS in stretched cells (15% isotropic for 30 minutes). While they saw an increase in CARTS, their membrane area increase was not significant. The authors conclude stretch induces a 12% increase in cell area. But in the graph (Figure 2E), this data is not significant- what is the area difference of each mean from each experiment? (each data set is a different colour Yellow/green/blue). How do the authors conclude there is an increase if the data is not significant? Doesn't this mean this is by chance and that the increase in CARTS is **not** due to an increase in area? In the JOVE article describing the stretch device, they find that focal adhesions on PAA gels only form with specific stretch cycles - although no detail is provided. Do focal adhesions also change with the stretch? They should be under more tension given the stretch is biaxial. Could this explain the increase in CARTS rather than the membrane increase?

Golgi tension data inconsistencies
The authors spread cells on FN and fixed cells over a time course, concluding that Golgi tension increases with cell spreading on FN. Again, the data appears to be different from t0 across the time course, but only for 2h and 4h. Oddly 3h is not significant. Again- micropatterning would be ideal for this. Plate cells on different areas and compare focal adhesion size, CARTS, Golgi tension and membrane area. Predicting that larger area would give you an increase in focal adhesions, CARTS number, and Golgi tension?
They compare Golgi tension on soft and stiffer PAA to Glass, while 0.2 kPa reduces Golgi tension, it is only significant when compared to glass. Yet- the authors conclude "FLIM measurements showed significantly lower lifetimes on soft substrates relative to stiff ones,". Is the 30 kPa not considered stiff in this context? Cells can spread on the 30 kPa and form focal adhesions, but not on 2 kPa (Fig S1F). This data would then suggest that this does not alter the Golgi tension? (i.e. Fig 3D 2 kPa vs 30 kPa, Golgi tension is not significantly different between these two conditions p0.1680 means there is a 16.8% probability that any difference between the conditions is due to chance).

The role of microtubule stability
For me, this part of the project as presented is somewhat disconnected from the rest. They abstractly rationalize that the microtubule sequestered Rho GEF, GEFH1, may be involved in this biology as GEFH1 activity is reported to activate PLCe>DAG>PKD. They chose to modify tubulin acetylation which controls GEFH1 association with microtubules. i.e microtubule acetylation promotes the release of GEF-H1 from microtubules into the cytoplasm. (I am not sure why they don't just directly test GEFH1? Or alphaTAT via shRNA).

They concluded FN spreading increased microtubule acetylation in comparison to PLL but, they do not observe the increase in tubulin acetylation with a static 15% strain. (microtubule acetylation increases with compressive stress, not strain, so this is not surprising doi: 10.1038/s41563-023-01578-1). I am not sure why they included this as their earlier strain data was inconclusive too. (which conflicts with their statement on line 24, "Collectively, these results indicate that both physiological spreading forces as well as externally applied stretch enhance MT acetylation"???)
They increase tubulin acetylation with tubacin (HDAC6i) and see an increase in CARTs but no increase in cell area. Was niltubacin considered as a control? As tubacin can result in an increase in peripheral focal adhesions, could this be why CARTS increase in the Tubacin treatments?

If CARTS export is blocked- can cells spread?
This was the most convincing data that Golgi trafficking is linked to spreading, but they fail to test the role of mechanics in this. They somewhat touch on this in the final figure showing when they inhibit PKD, they saw less CARTS with a decrease in area with a corresponding decrease in Golgi tension. They also specifically inhibit CARTS using a TGN46 KO, and saw a significant decrease in cell spreading. They should validate that there are less CARTS in this condition when cells spread. If integrin activation and FA formation require CARTS, is the converse true? Have the authors considered what happens if you block CARTS to FA size and integrin activation, given cells do not spread without CARTS

Minor
(1) Figure 1 is mislabeled:
(C), the adhesion surface area per cell (D), and the number of CARTS per unit adhesion area
They are labelled the other way around on the figure.
(2) Figure s4- please display the images using the same scale bars, particularly A DMSO 1h vs 4 h- this will make sure the reader understands that the size increase matches the increase in area shown in the graph).
(3) They say no FA are formed on PLL at 4 hours, but do not show the control FN cell to show FA on FN at 4h
(4) information on statistical methods used was lacking, more detail would be helpful.

Reviewer #1 (Comments to the Authors (Required)):

The authors explore the potential connection between mechanical forces and Golgi complex function, particularly in the generation of vesicles destined for the plasma membrane. This is an intriguing topic, as the Golgi apparatus remains relatively understudied in the context of mechanobiology. Although previous studies have shown that the Golgi is a mechanosensitive organelle that responds to factors such as matrix stiffness and cell adhesion, a direct link between mechanical forces and post-Golgi carrier formation has not yet been established. This work therefore addresses an important and timely question, how the Golgi apparatus functions as a mechanosensor that coordinates exocytic trafficking with focal adhesion (FA) assembly and cell adhesion, revealing potential positive feedback mechanisms within this process.
The experiments are well designed and carefully executed, with clear data presentation and appropriate statistical analyses. However, while the authors clearly demonstrate that Golgi carrier biogenesis is linked to mechanical forces, the manuscript currently lacks a clear mechanistic link connecting the observed phenomena. Additional work is needed to establish how the identified processes are causally related.

We thank the reviewer for their positive assessment of our work and for carefully reading the manuscript. We also appreciate the balanced and constructive comments and suggestions provided. Our detailed responses to the reviewer's points are given below.

General comments:
The DAG reporter used is based on the C1 domain of PKD. However, this construct may compete with endogenous PKD for DAG binding, potentially influencing CARTS formation. Moreover, given the feedback loop between active PKD and DAG generation (as shown by Fugmann et al. 2007), the reporter may obscure the true dynamics of DAG levels. As an alternative, the authors can employ alternative reporters that directly monitor PKD activity at the Golgi (e.g. using a genetically encoded PKD reporter), as this would more accurately reflect DAG-related generation of CARTS.

We thank the reviewer for raising this important point. We agree that precise, quantitative measurement of DAG levels (and in general, of any particular lipid species) at the Golgi/TGN membranes remains technically challenging, in part because most available probes are derived from lipid-binding domains in functional proteins (such as the PKD-C1a domain used here). As the reviewer notes, these probes have the general limitation that they may potentially interfere with endogenous protein-lipid interactions and could therefore influence the processes being studied. We had considered the use of alternative DAG probes. However, after evaluating the available sensors and discussing this with colleagues who are experts in DAG and lipid reporters, we concluded that other probes would likely present similar limitations to those associated with the PKD-based reporter used in our study. We therefore followed the reviewer's suggestion and focused on directly monitoring PKD activity at the Golgi/TGN (see below).

But before describing those new experiments, we would like to share with the reviewer some of our unpublished observations, which, although not conclusive, suggest that the PKD-based DAG reporter we have used may not significantly compete with endogenous PKD during CARTS biogenesis. PKD is a multidomain protein containing (i) an N-terminal ubiquitin-like domain required for homo-/hetero-dimerization (Elsner et al., PMID: 31406020,

2019), followed by (ii) the C1a domain responsible for DAG binding (i.e., our DAG sensor), (iii) a C1b domain involved in Arf1 binding, (iv) an autoinhibitory PH domain, and (v) the catalytic kinase domain (see e.g., Wakana and Campelo, PMID: 34203456, 2021 for further details). Kinase-dead PKD mutants (PKD-KD) act as TGN-localized dominant-negative inhibitors of endogenous PKD and inhibit TGN export. Our data indicates that this dominant-negative effect of PKD-KD is because it dimerizes with endogenous PKDs, as a non-dimerizable kinase-dead mutant (PKD2-Δ1–137-KD), which still localizes to the TGN and retains DAG-binding capacity does not interfere with the export of the CARTS-dependent cargo ss-HRP (**Fig. R1**). Based on these observations, we expect that expression of the isolated C1a domain (which binds DAG but lacks kinase activity and cannot dimerize with endogenous PKD) would similarly not act as a dominant-negative construct, and hence endogenous PKDs could still be recruited to the TGN by DAG binding and promote CARTS fission.

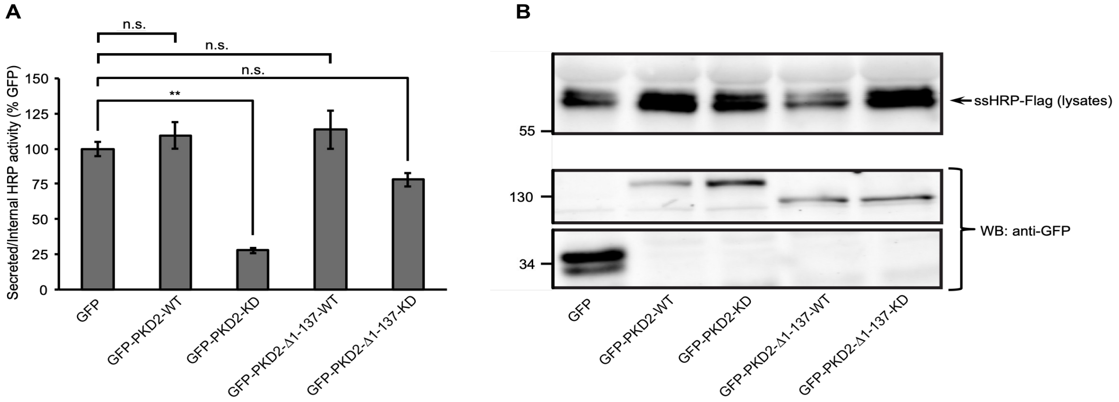

Fig. R 1. PKD dimerization is required for inhibition of CARTS-mediated cargo export.
HeLa cells stably expressing ssHRP-Flag were transiently transfected with the indicated GFP-tagged constructs. ***(A)*** *Secretion of ssHRP (for 4 hours) was quantified by electrochemiluminescence and normalized to the internal pool. Expression of kinase-dead PKD (GFP-PKD2-KD) strongly inhibited ssHRP secretion, whereas a non-dimerizable kinase-dead mutant (GFP-PKD2-Δ1-137-KD) did not significantly affect cargo export. Data are shown as mean ± SEM (n=3) normalized to the GFP control; **p < 0.01; n.s., not significant, One-way ANOVA.* ***(B)*** *Immunoblots show ssHRP-Flag expression levels (lysates) and expression of GFP-tagged constructs (anti-GFP).*

Nevertheless, we agree with the reviewer that directly assessing PKD activity would provide a robust, complementary approach. We therefore monitored PKD activity at the Golgi/TGN using the genetically encoded fluorescence reporter G-PKDrep, developed by the Hausser lab (see e.g., Fuchs et al., PMID: 19416469, 2009; Eisler et al., PMID: 30028295, 2018). This reporter consists of EGFP fused to the PKD substrate motif of PI4KIIIβ together with a TGN-targeting sequence (the GRIP domain of p230) to target the reporter to the TGN. PKD activity is quantified by detecting phosphorylation of the substrate motif using a phospho-specific antibody (pS294) and normalizing the signal to the EGFP fluorescence signal (reporter expression). Using a HeLa cell line stably expressing doxycycline-inducible EGFP-PKDrep (kindly provided by Prof. Angelika Hausser, University of Stuttgart), we found that PKD activity at the Golgi is higher at early time points after cell seeding on fibronectin (FN), consistent with the increase in DAG levels previously observed using the C1a reporter (new Fig. 6). Interestingly, we also observed a detectable increase in PKD activation at early time

points in cells seeded on PLL, which was somewhat unexpected given that PLL-seeded cells showed low Golgi DAG and reduced CARTS biogenesis. Additional experiments will be required to determine whether this acute Golgi-localized PKD activation reflects a DAG-independent input, a transient DAG signal not captured by our assay, or another adhesion-independent signaling mechanism.

In the revised manuscript, we have first expanded the discussion of the DAG reporter data to explain better the limitations of C1 domain-based probes, including the explanation of the feedback loop between PKD activation and DAG production. We then present the new measurements of PKD activity using the genetically encoded reporter (new **Fig. 7***) as a new main figure. We considered moving the DAG data to the supplementary material, but ultimately decided to keep them as a main figure (***Fig. 6***) due to the limit on the number of allowed supplementary figures (maximum of five).*

Taken together, we think that these combined datasets provide a consistent picture, although we interpret them with appropriate caution given the limitations of the available tools. Further work will be required to more precisely dissect the underlying mechanisms.

The molecular mechanism linking microtubule (MT) acetylation to DAG levels at the TGN remains insufficiently explored. The authors propose involvement of the GEF-H1/Rho pathway, and indeed, a recent preprint suggests a connection between this pathway and MT acetylation (Roy et al 2024, https://doi.org/10.1101/2024.12.01.626286). Testing the effects of Rho inhibition/activation on CARTS formation and Golgi-dependent exocytic activity would significantly strengthen this hypothesis. Alternatively, employing Rho or GEF-H1 activity reporters could help determine whether Golgi-mediated exocytic activity provides feedback to this signaling pathway.

We thank the reviewer for this insightful comment and for pointing us to the recent work by Roy et al. (bioRxiv 2024; recently published in its final form in JCB, https://doi.org/10.1083/jcb.202508095), which we find highly relevant. This study introduces an optogenetic tool (optoTAT) to locally induce microtubule acetylation and provides direct mechanistic evidence that microtubule acetylation promotes GEF-H1 release for subsequent RhoA activation, thereby strengthening the pathway we propose. We have incorporated a discussion of this work in the revised manuscript. In the future, it will be particularly interesting to exploit this approach to directly test key predictions of our model with respect to the role of microtubule acetylation in carrier biogenesis at the TGN.

We complemented our analyses by assessing downstream readouts of Rho signaling. We first focused on Myosin light chain (MLC) phosphorylation (at Ser19) as a reporter of Myosin II activation downstream of Rho/ROCK signaling (new **Fig. S3***). In our previous work (Miserey-Lenkei et al., PMID: 20562865, 2010; Miserey-Lenkei et al., PMID: 29093437, 2017), we showed that phosphorylated MLC (pMLC) associates with RAB6-positive carriers at the Golgi and that Myosin II activity (inhibited using the ROCK inhibitor Y-27632 and MLCK inhibitor ML-7) is required for the biogenesis and fission of post-Golgi carriers at the TGN membrane. Consistent with this, and in line with our results using the DAG and PKD activity reporters, our new data show that pMLC levels per cell peak at early time points (1h) after seeding on FN and then return toward later steady-state levels at 4h and 24h. Although additional experiments will be needed to fully establish the significance of this transient increase, these results are consistent with our proposal that Rho-dependent signaling is enhanced at early stages of cell spreading on FN. In parallel, phalloidin staining confirmed*

the formation of stress fibers in cells seeded on FN, in contrast to cells seeded on PLL, suggesting active Rho in these conditions. Moreover, previous work has shown that human fibroblasts plated on FN display a faster and stronger increase in phosphorylated MLC during spreading compared to PLL (Ren et al., PMID: 15226371, 2004), consistent with differential activation of contractility pathways under these conditions.

Importantly, previous work (Zilberman et al., PMID: 21680709, 2011) demonstrated that the formation of RAB6-positive carriers at the TGN is enhanced by constitutively active RhoA (RhoA-V14 mutant) expression, supporting a functional link between Rho signaling and post-Golgi trafficking. This effect, which was shown to depend on formin activity, is consistent with a role for Rho-mediated cytoskeletal regulation in Golgi-derived carrier biogenesis.

Taken together, our data suggest that early cell spreading on FN triggers a transient Rho/ROCK-dependent program that couples actomyosin contractility to Golgi secretory activity, as reflected by the early increase in pMLC, DAG, and PKD activity and consistent with a role in rapid membrane remodeling and Golgi-derived carrier biogenesis. Although the precise causal relationships between microtubule acetylation, Rho signaling, and DAG regulation at the TGN remain to be established, these results support the view that these pathways are functionally linked. We have revised the manuscript accordingly to keep this interpretation appropriately cautious, and further work, particularly using more specific activity reporters for Rho or GEF-H1, will be needed to dissect the underlying mechanisms.

In addition to pharmacological inhibition with tubacin, employing hypo- and hyper-acetylated microtubule (MT) constructs (Joo et al., 2014) could offer more direct evidence connecting MT acetylation to Golgi-dependent exocytic function, particularly given that HDAC6 acts on multiple substrates beyond tubulin.

We thank the reviewer for this valuable suggestion. We agree that the use of hypo- and hyper-acetylated microtubule constructs would provide a more direct means to assess the role of microtubule acetylation, particularly given the pleiotropic effects of HDAC6 inhibition with tubacin (also noted by Reviewer #3). However, following the editor's guidance, we have prioritized other experiments in the current revision and plan to pursue this line of investigation in future studies. We have nonetheless revised the manuscript to explicitly acknowledge this limitation and to clarify the interpretation of our results.

Finally, it would be important to assess the impact on focal adhesion dynamics (in addition to cell adhesion) when Golgi exocytic activity or MT acetylation is inhibited. This could help define the feedback relationship between Golgi mechanosensing, vesicle trafficking, and adhesion assembly.

We thank the reviewer for this important point, which was also raised by Reviewer #3 (see response to Reviewer #3). Following this suggestion, we examined the impact of inhibiting Golgi exocytic activity on focal adhesions (FAs). Specifically, we seeded cells on FN for 1h or 4h in the presence of the PKD inhibitor CRT0066101 or its vehicle (DMSO). Our new results indicate that PKD inhibition, which prevents total cell spreading after 4h (see **Fig. 8D***), does not cause a substantial change in the levels of active β1 integrin at the cell surface at either 1h or 4h after seeding (new* **Fig. S5C, D***). By contrast, the FA area is reduced in cells treated with the PKD inhibitor for 4h compared with control cells (new* **Fig. S5E***), consistent with the correlation between FA area and overall cell spreading area.*

Interestingly, previous work (Eisler et al., PMID: 30028295, 2018) reported no changes in the number of FAs per cell in cells depleted of both PKD2 and PKD3 by siRNA. These observations suggest that inhibition of Golgi exocytic activity primarily affects overall cell spreading, with secondary consequences on FA area, rather than causing a major defect in β1 integrin activation per se. These results have been incorporated into the revised manuscript and discussed accordingly.

Specific comments:
Figure 3B: The observed drop in Halo-Flipper lifetime at 3 hours post-seeding is unexpected, especially since the lifetime increases again at 4 hours. The authors should provide an explanation for this transient decrease, as it seems inconsistent with the overall trend.

We indeed agree that the observed drop in Halo-Flipper lifetime at 3 hours post-seeding is unexpected given the overall trend. Flipper probes are among the most sensitive and informative reporters of membrane tension and lipid packing when used sensibly. However, their performance in cells (particularly for Halo-conjugated Flipper variants) is still challenging and can certainly suffer from cell-to-cell and experiment-to-experiment variability.

To improve the robustness of our analysis, we have included an additional independent biological replicate (this time with ≥30 cells per condition, to ensure enough cell-to-cell variability sampling within this replicate). With this expanded dataset, the 3h value now appears to be statistically significantly higher than the earliest time point (0.5h), with a p-value <0.05. Interestingly, we can still qualitatively see a slight decrease in Flipper lifetime at the 3h time point relative to neighboring time points. We note that in this additional replicate, the 0.5h time point had to be excluded due to very poor Halo-Flipper labelling (almost no signal detected), which rendered the cells unsuitable for imaging. After consultation with statistics experts, we proceeded with the analysis, as the exclusion was due to a random technical issue (i.e., a "bad" sample). As a consequence of the missing value, the dataset could not be analyzed using repeated-measures ANOVA. Instead, we applied a mixed-effects model (REML), which allows for missing data points, assuming sphericity and matching across biological replicates (see new ***Fig. 3D****).*

Overall, while the mean Flipper lifetime at 3h appears slightly lower than at other intermediate time points, it remains significantly higher than in the initial condition. The updated results and corresponding discussion have been revised accordingly in the manuscript.

Figure 4B: Please include the p-values for the PLL group to allow proper statistical evaluation and comparison across conditions.

To address this point, we reanalyzed the dataset using a two-way ANOVA with substrate ligand (FN or PLL) and time (1h, 4h, 24h) as factors, rather than the previous one-way ANOVA in which all conditions were treated as independent groups (see new ***Fig. 4B****). The original one-way ANOVA we believe was appropriate for the specific set of comparisons initially considered, under the assumption that each condition was being evaluated as a separate group. However, because the revised analysis suggested by the reviewer explicitly aims to compare responses across both time and substrate within the same experimental design, a two-way ANOVA is the more appropriate method. The new analysis is shown in*

the revised figure and supports our original conclusion: acetylated tubulin levels increase significantly over time in cells seeded on FN, whereas no significant time-dependent change is detected in cells seeded on PLL.

Figure 6A and B: The reported decline in DAG levels measured with the Golgi sensor does not appear to correlate with the observed increase in CARTS formation shown in Figure 1. The authors propose that this may result from DAG incorporation into nascent transport carriers or conversion into other lipids required for membrane fission. However, if DAG is being depleted, it is unclear how CARTS formation remains robust (Fig. 1C). Similarly, treatment with tubacin enhances CARTS formation without affecting DAG levels. These discrepancies suggest that DAG quantification through the current sensor might not fully capture functionally relevant DAG dynamics.
As previously mentioned, employing a PKD activity reporter could provide a more direct and reliable measure of CARTS formation, given that PKD activation is tightly coupled to this process.

As discussed above, we fully agree that DAG quantification using the current Golgi-targeted sensor has important limitations and may not be sufficiently sensitive to capture the functionally relevant dynamics of DAG during CARTS formation. We have now included the new data using a PKD activity reporter as a main figure (new **Fig. 7***) and move the DAG data as supplemental material (***Fig. 6***). Interestingly and in accordance with our previous DAG data, PKD activity seems to peak at early timepoints while CARTS production continues to increase in later timepoints. Our interpretation is that early increase in DAG synthesis at the Golgi and localized PKD activation are the trigger for generation of CARTS which persists longer in time even when the signaling has been attenuated at 4 hours, which could explain why CARTS formation plateaus at 4h.*

*We would nonetheless like to provide a clearer explanation of how we interpret the relationship between DAG levels (***Fig. 6B***) and CARTS formation (***Fig. 1C***). We understand our data not as an indication of a "depletion" of DAG at later time points (4h, 24h), but rather a transient "increase" at early stages (at least for the 1h time point we measured). We speculate that this early increase in DAG levels may act as a trigger that promotes subsequent CARTS biogenesis, while steady-state DAG levels at later time points remain sufficient to sustain basal carrier formation. That being said, we cannot ignore the limitations of the DAG sensor, which reports bulk DAG levels at the Golgi but may not distinguish localized and/or transient DAG pools that are functionally engaged in carrier biogenesis. DAG is likely to be rapidly turned over during CARTS biogenesis, either through incorporation into nascent carriers, as proposed, and/or by conversion into other lipid species involved in membrane fission (such as PA, LPA, see e.g., Campelo and Malhotra, PMID: 22463692, 2012). As a result, global steady-state DAG levels may not accurately reflect its functional availability at specific CARTS fission sites.*

Similarly, the observation that tubacin treatment enhances CARTS formation without detectable changes in DAG levels further supports the idea that DAG-dependent processes may be regulated at spatial and/or temporal scales not captured by the sensor. Alternatively, it is also possible that additional pathways act in parallel to DAG signaling to promote CARTS biogenesis. Our revised text has been expanded to briefly include these points of discussion.

Figure 7: It would be highly interesting to test whether PKD inhibition, particularly under conditions where cells are plated on fibronectin, affects microtubule (MT) acetylation. Such an experiment could provide valuable insight into the feedback relationship between CARTS formation and MT acetylation discussed earlier.

We thank the reviewer for this insightful comment. We have now performed the suggested experiment to assess whether PKD inhibition affects MT acetylation in cells plated on FN. Our results indicate that treatment with the PKD inhibitor CRT0066101 for 4 h does not lead to a significant change in acetylated tubulin levels relative to total tubulin (new ***Fig. S5F,G****).*

While additional experiments would be required to fully address this question, our new data are consistent with the proposed model in which MT acetylation acts upstream (via GEF-H1 release and Rho activation) of PKD activation. They further suggest that any potential feedback from PKD-dependent processes, such as CARTS formation, is not strong enough (at least to the level of our experimental sensitivity) to modulate global MT acetylation levels during cell spreading.

*These new data have been included in the revised manuscript (****Fig. S5F,G****), and the corresponding discussion has been updated accordingly.*

Reviewer #2 (Comments to the Authors (Required)):

In this paper, the authors show the novel finding that the Golgi apparatus functions as a mechanoresponsive organelle whose secretory output is directly modulated by extracellular mechanical forces. This work establishes a mechanotransduction axis linking the extracellular matrix to directed Golgi-to-plasma membrane (PM) trafficking.
The study demonstrates that mechanical input-specifically increased substrate stiffness, robust cell spreading, or applied equibiaxial strain-significantly enhances Golgi transport carrier biogenesis, notably increasing the exocytosis of CARTS (carriers of the TGN to the cell surface). This augmented Golgi export is essential for cell adaptation, as its inhibition (via Golgicide A or PKD inhibition) or the depletion of the CARTS adaptor TGN46 severely impairs sustained cell spreading and mechanoadaptation.
The paper elucidates a specific molecular and biophysical cascade transducing the mechanical signal:
-Membrane Mechanics. Extracellular forces correlate with a progressive increase in Golgi membrane tension.
-Cytoskeletal Link. Robust spreading enhances microtubule (MT) acetylation, a modification that is sufficient to promote CARTS biogenesis independently of external force when experimentally mimicked (e.g., using tubacin).
-Signaling Axis. MT acetylation feeds into the RhoA/PLCε/DAG/PKD axis, leading to the accumulation of diacylglycerol (DAG) at the Golgi. DAG-mediated activation of Protein Kinase D (PKD) is identified as necessary for the subsequent carrier formation and membrane remodeling.
The authors propose a comprehensive model where forces transduced from focal adhesion (FA) establishment promote MT acetylation, which then activates the RhoA/PLCε/DAG/PKD pathway to boost Golgi export, thus enabling cellular adaptation to its mechanical environment.

We thank the reviewer for carefully reading of our paper and for providing positive and constructive assessment of our work.

Response to Minor Revisions
The following points represent necessary clarifications and data presentation checks required to finalize the manuscript.
-It is noted that while spinning-disk microscopy was conducted at 37 the temperatures for Fluorescence Lifetime Imaging Microscopy (FLIM) and Live-cell TIRF measurements are not explicitly stated.
-Please clearly specify the temperature used for all FLIM and Live-cell TIRF experiments.
-Given that the study focuses heavily on membrane mechanics and lipid dynamics (DAG levels and Golgi membrane tension via Halo-Flipper), it is critical to confirm the temperature. Lipid behavior, including membrane order and phase separation, is highly sensitive to temperature changes, and failure to control or report the temperature, especially between room temperature 37 could introduce confounding variables or make lifetime data incomparable.

We appreciate the reviewer's comments on these important points. We apologize for the omission and fully agree with the reviewer that temperature control is critical, in particular for experiments involving membrane mechanics and dynamics. We performed all live-cell

imaging experiments, including spinning-disk, confocal microscopy and FLIM, at 37 ºC. This information is now explicitly stated in the revised text.

-Please ensure that the total number of independent experiments N and the total number of cells or measurements n are clearly provided for the following figures: Figure 1H, Figure 3B/D, Figure 5F, Figure 7F, Figures S2B, and S3B.

We have carefully revised all relevant figure legends to ensure that both the number of independent experiments (n) and the minimum number of measurements or cells (N) per each individual biological replicate are clearly indicated.

-Please clarify precisely what each individual point and/or colored data group on the scatter plots and bar graphs represents (e.g., is each point the median value derived from one cell, or an aggregate measurement from a population of cells, or a biological replicate. This detail is crucial for interpreting the statistical methodology.

We thank and fully agree with the reviewer and have now systematically added this information in the revised version. We have also expanded the Methods section under "Statistical analysis and data representation" to make sure that the graphical representation of the data is clear and readily interpretable.

We would like to stress that we chose to display many of our datasets using SuperPlots (see Lord et al., PMID: 32346721, 2020), as we think this is the most correct way to represent experimental cell biology data while visually conveying both cell-to-cell variability (different cells measured within a single biological replicate) and experiment-to-experiment reproducibility (day-to-day variability among independent biological replicates). As emphasized by Lord et al., statistical descriptors (such as P values and SEM) should, in most cases, be calculated using biological replicates (i.e., independent measurements obtained from separate experiments) rather than treating each individual cell as an independent n, which can artificially inflate significance.

In brief, in our SuperPlots, each small, light-colored symbol represents one individual cell measurement, whereas the larger, black-outlined colored symbols represent a central value (typically the mean) of all cells (N) measured within one independent biological replicate. Different colors denote different biological replicates (n). Horizontal black lines and error bars indicate the mean ± SEM across biological replicates, and the corresponding statistical tests were performed using the biological replicate means as n (rather than the total number of measured cells).

We fully agree that these details are essential for proper interpretation of the statistical analyses, and we have now clarified them systematically in the figure legends and Methods section.

Reviewer #3 (Comments to the Authors (Required)):

This work builds on publications describing exocytosis occurs near FAs occurs at exocytic hotspots (a term coined by Yau et al MBoC 2011) spatially controlled by microtubule targeting (first reported in Stehbens et al NCB 2014 identifying Rab6 exocytic hotspots at FA) which coordinates post Golgi secretion at these PM sites (Fourriere et al., 2019).

Here they explore the idea of mechanochemical feedback loops between adhesion, membrane tension (focusing on Golgi but PM would also be implicated) with Golgi-derived biogenesis and exocytosis via mechanical stimuli. While we know a lot about focal adhesions in sensing mechanical signals, there is less known about organelles like the Golgi. (although there has been a significant shift in our knowledge of nuclear mechanosensing too- I would suggest adding a sentence referring to this near line 6-9 where organelle mechanosensing is first introduced).

I wholeheartedly agree- there is likely a mechanochemical feedback loop linking microtubules, and membrane tension with targeted exocytosis at focal adhesions which enables cells to modify their membrane tension. Golgi membrane mechano-sensitivity will be key to understanding this biology. However, while interesting, the data as presented fails to convincingly connect the phenomena and is largely speculative. They often equate results with biological phenomena that they do not directly test. Several of their experimental manipulations alter CARTS -but do not affect the membrane. I strongly suggest they focus on focal adhesions in this context as they might be the link they are missing in their data. I did enjoy the discussion where they expand on their ideas about membrane exo-endo cytosis balance and their identification of some tool shortfalls- particularly the issue of Flipper probes in Golgi tension measurements. I outline several queries below in more detail which I think would help the authors to better articulate their work in a more comprehensive way. I hope that the authors find this helpful.

We thank the reviewer for their careful, thoughtful, and constructive assessment of our work, and for the many interesting ideas and suggestions provided. We particularly appreciate the reviewer's perspective on the potential existence of mechanochemical feedback loops linking focal adhesions (FAs), membrane tension, cytoskeletal dynamics, and localized exocytosis, which indeed are at the core of the main ideas we are currently investigating.

We also note that pioneering work from others (including Grigoriev et al., PMID: 17681140, 2007; Stehbens et al., PMID: 24859005, 2014) established the concept of microtubule-guided exocytosis at/near FAs. In the revised manuscript, we have expanded the introduction to better reflect this body of work, including earlier references related to the concept of "exocytic hotspots" and spatially organized secretion (e.g., Robinson et al., PMID: 7708668, 1995; Yuan et al., PMID: 25606674, 2015).

We fully agree with the reviewer that mechanosensing is not limited to FAs and the Golgi, and that other organelles, most notably the nucleus, are now recognized as key players in cell mechanotransduction. In response to this suggestion, we have added references to recent reviews on nuclear mechanosensing in the introduction to place our work in a broader cellular context.

We also understand the reviewer's concern that some aspects of the original manuscript may have appeared too speculative. In the revised version, we have more clearly distinguished between data-supported conclusions and interpretative/speculative elements

of our model. In addition, following the reviewer's suggestion, we have added new data on FA and integrin-dependent processes (see detailed responses below).

Overall, we believe that these revisions have improved the clarity and rigor of our manuscript and have helped us ensure that our conclusions are more appropriately supported by the data.

***CARTS exocytosis at adhesions*.**
They first image CARTS exocytosis at adhesions via TIRF. They state that the PAUF positive carriers (to mark CARTS) exit the Golgi and dock at FA. To better support this conclusion, I would encourage the authors to quantitate this data- it is live imaging, it should be relatively straight forward to measure proximity relative to FA over time, as well as dock, dwell and fusion measurements. (Although I did see the authors chose to only image focal adhesions once over the 45 min duration).

We agree with the reviewer that having such a full quantitative analysis of CARTS exocytosis would have provided valuable additional information. Unfortunately, due to the configuration of our TIRF microscopy setup at the time of acquisition, FAs were imaged –as the reviewer noticed– only once as a single snapshot, which was used to generate a mask for the entire duration of the time-lapse. As a result, quantification of parameters such as distance to FAs over time would not have been reliable. For this reason, we only provided a simple quantification of these data in the original submission (former Fig. S1C; now ***Fig. S1E,F****). This plot (***Fig. S1F***) shows how the spatial distribution of mKate2-FM4-PAUF cargo intensity relative to the FA mask changes over time after cargo release from the ER. When comparing the two time points, 20 min, when the cargo is mainly Golgi-localized, and 40 min, when the cargo is predominantly in post-Golgi carriers en route to the PM, we observed a shift in the histogram toward shorter nearest-neighbor distances to FAs. This shift quantitatively indicates that post-Golgi PAUF-positive carriers become progressively enriched near FA regions.*

We also note that a more rigorous analysis of exocytic events would benefit from improved reporters (e.g., PAUF fused to a pH-sensitive fluorophore such as pHluorin), which would allow a clear distinction between bona fide exocytic events and other signal losses (e.g., photobleaching or out-of-focus movement). Because the preferential targeting of different Golgi-derived carriers (including RAB6 carriers, integrins, etc.) to FA-rich regions of the plasma membrane is well established (as also noted by the reviewer), we thought it was best to prioritize other experiments.

Nonetheless, to further support this aspect of our work, we have now investigated whether the spatial distribution of CARTS (which represent a subset of RAB6-positive TGN-to-PM carriers) is similarly affected by depletion of ELKS, a protein involved in the spatial organization of exocytosis at the plasma membrane, as reported in our previous work where we have shown the ELKS- and RAB6-dependent arrival of secreted cargos at secretion hotspots (Fourriere, Kasri, et al. PMID: 31142554, 2019). Our new results show that ELKS knockdown leads to an accumulation of CARTS in regions of the cytoplasm proximal to FAs (marked by paxillin), consistent with previous observations for RAB6 carriers. These new data are now included in ***Fig. S1B-D*** *and further support the conclusion that CARTS are preferentially targeted to FA-rich regions.*

They then compare CARTS # in cells seeded on PLL vs FN coated substrates. They

compare membrane area to CARTS # on the two substrates, concluding that FN attached cells have more CARTS and an increase in area. Q: Is the total surface area of the cell smaller overall? i.e. are the PLL cells not spread, but are higher? A z stack should be able to demonstrate this.

We thank the reviewer for raising this interesting point. We fully agree that projected adhesion area does not reflect total plasma membrane area nor full 3D cell morphology, as less spread cells can be "taller". Accurately measuring total plasma membrane surface area in 3D is technically challenging, as it would require accounting for membrane reservoirs such as folds, tubules, caveolae, etc. Moreover, although the z-stacks used in Fig. 1D are suitable for quantifying CARTS per cell, they were not acquired to fully reconstruct cell volume, and we therefore prefer not to overinterpret them for this purpose. We have revised the manuscript to clarify that our measurements refer to projected adhesion area rather than total cell surface area or volume. We also now discuss this in light of previous work showing that differences in spreading are not simply compensated by increase cell height at constant size, but can also be accompanied by changes in cell volume itself. In particular, Venkova et al. (PMID: 35416768, 2022) showed that HeLa cells spreading on FN undergo a transient loss of volume (during the initial ~30 min), whereas non-spreading cells plated on PLL-PEG increase their volume over time, suggesting that spreading dynamics are coupled to volume regulation. Likewise, Guo et al. (PMID: 28973866, 2017) showed that cells on stiffer substrates display reduced volume compared with cells on softer substrates, and that restricting spread area increases both cell height and cell volume. Thus, while our data support a correlation between projected adhesion area and CARTS number, they do not allow us to determine whether total plasma membrane surface area or cell volume differs between FN- and PLL-seeded cells.

The role of integrin activation

The authors conclude/infer that integrin activation and focal adhesion formation act to coregulate Golgi export- however, do not test any effects on focal adhesion formation nor integrin activity.

They look at focal adhesion formation on 2 vs 30 kPa gels (vinculin) and conclude that as focal adhesion numbers are less, the decrease in CARTS is due to modification in integrin activation (but do not test integrin activity directly). Cell morphology is not always an indicator of integrin activation - see this work where stem cells on soft substrates had higher integrin B1 activation. This requires specific antibodies which should be able to detect the total and **activated** to support their statement

https://www.pnas.org/doi/10.1073/pnas.1106467108

If they add +Mn or cRGD to modify integrin activation, does this alter CARTs biogenesis?

We thank the reviewer for this important point and we now realize that our original wording overstated the link between integrin activation, FA formation, and Golgi export, which was not our intention. We have now carefully revised the manuscript to clarify that our data support the link between adhesion-dependent cell states and Golgi export, but do not directly demonstrate a causal relationship between integrin activation at FAs and CARTS biogenesis, which was not the primary aim of this work.

To provide some insights into this interesting possibility, and in response to the reviewer's suggestion, we have now included new experiments assessing integrin β1 active state using a conformation-specific antibody (9EG7 clone). Our new results show that indeed, as

*predicted by the reviewer, we observed no statistically significant differences in active β1 integrin levels in cells seeded on FN-coated glass compared to cells seeded on softer substrates (***Fig. S2D,E***). What is different is the surface area covered by FAs per cell, which appears to be smaller in cells seeded of soft substrates vs. glass (***Fig. S2D,F***). These data show positive correlation between FA area, but not active β1 integrin levels at the cell surface, and the number of cytoplasmic carriers. Moreover, we agree that directly probing the role of integrin activation and downstream signaling in Golgi-derived carrier formation will be important. Approaches such as Mn^{2+} or cRGD treatment, as suggested by the reviewer, would provide a more direct test of this hypothesis; however, we consider these experiments beyond the scope of the current revision. We have nonetheless included this point in the discussion as an important direction for future work and we have deleted all reference to integrin activation from the text to avoid misinterpretation.*

CARTS and mechanical signals
The authors propose that CARTS export might be mechanically linked. They use tuned substrates and strain. The results are often variable, which suggests maybe they need to consider additional control of the mechanical signal to rule out confounding effects. Here, I think micropatterning would be very effective. Can you tune CARTS export and number by plating cells on micropatterned substrates of various sizes? This could be combined with PAGs and adhesion substrates to control membrane tension associated with spreading. See https://doi.org/10.1242/jcs.075150

We truly thank the reviewer for this valuable suggestion and for pointing us to relevant literature. We agree that micropatterning approaches would provide a powerful and well-controlled way to modulate cell shape, adhesion, and, ultimately, mechanical inputs, and could help reduce variability and more precisely dissect the relationship between mechanical cues and CARTS biogenesis/Golgi export in general. However, following the editorial advice, we have prioritized on other experiments and not pursued this approach for the current paper. Instead, we have carefully revised the manuscript to acknowledge that variability in our measurements may partly reflect the limited control over mechanical inputs in our experimental setups. We now explicitly discuss this limitation and highlight micropatterning-based strategies as an important avenue for future work (which we will certainly test in the future!)

Confounding data lacking significance
They compare CARTS in stretched cells (15% isotropic for 30 minutes). While they saw an increase in CARTS, their membrane area increase was not significant. The authors conclude stretch induces a 12% increase in cell area. But in the graph (Figure 2E), this data is not significant- what is the area difference of each mean from each experiment? (each data set is a different colour Yellow/green/blue). How do the authors conclude there is an increase if the data is not significant? Doesn't this mean this is by chance and that the increase in CARTS is **not** due to an increase in area? In the JOVE article describing the stretch device, they find that focal adhesions on PAA gels only form with specific stretch cycles - although no detail is provided. Do focal adhesions also change with the stretch? They should be under more tension given the stretch is biaxial. Could this explain the increase in CARTS rather than the membrane increase?

Regarding the measurement of the adhesion area, the point raised by the reviewer is fair. In our stretching device, we apply equibiaxial strain to the PDMS substrate to which the cells adhere. Such substrate deformation leads therefore to an expansion of the adhesion area at early times after stretch initiation. However, cell-substrate adhesions are dynamic, so maintaining a constant strain for long times leads to the dynamic readjustment of their adhesion sites, which may ultimately lead to adhesion area relaxation. As the experimental design requires that we keep the constant strain for 30 min (as we needed some time for TGN export to resume after the 20ºC block), we hypothesize this is why we are not observing a statistically significant change in the adhesion area, as during that time it has already been at least partially readjusted.

As requested by the reviewer, we provide here the mean adhesion area values per condition (with and without stretch), as well as the relative changes observed across experiments:

**Area (Control) / um^2**	**Area (15% stretch) / um^2**	**Rel. Increase (%)**
1123	1378	22.70703473
982.4	1123	14.31188925
1033	1065	3.097773475

We would nonetheless like to emphasize that, throughout the manuscript, the data are displayed and analyzed using a SuperPlots framework (please see also our response to Reviewer #2). Although this type of representation is still not the standard procedure, we consider it the most appropriate way to convey both cell-to-cell variability and experiment-to-experiment reproducibility. As a consequence, the statistical significance may appear more modest than expected when compared with analyses that treat each individual cell as an independent n. In our analysis, statistical testing was performed using the means of the biological replicates (here, n = 3 independent experiments), rather than pooling all measured cells across replicates (approximately N = 150) into a single test. Indeed, if all individual cells were analyzed together using a two-tailed unpaired Student's t-test, the resulting P value would be ~ 5×10^{-8}. However, while this latter approach is common in the literature, and can be informative in some contexts, we felt that, for the present study, analyzing biological replicates as the independent n provides a more appropriate representation of experimental reproducibility and variability between independent experiments.

Regarding the JOVE article, the reviewer is correct that only limited details are given in there. The figure in that paper illustrates the possibility of combining the stretching device with tunable substrate rigidity (using PAA gels attached to the PDMS membrane), and refers to previous work from one of our groups (Andreu, Falcones, et al., PMID: 34244477, 2021), showing that, in that specific configuration, FA growth and reinforcement depend strongly on the loading rate imposed by cyclic stretch. Only certain cyclic stretch regimes promoted adhesion reinforcement. The experimental setup/design is different. Here, cells were seeded directly on FN-coated PDMS, which is much stiffer (MPa range) and supports robust adhesion. Moreover, we applied a single and sustained 15% equibiaxial stretch for 30 minutes, rather than cyclic stretch. Under these conditions, FAs may rearrange, but not in the same way as in the PAA-based cyclic stretching experiments. We therefore agree with the reviewer that changes in FA tension and mechanotransduction could contribute to the

increase in CARTS, in addition to any effects on membrane tension. We have revised the text accordingly to better reflect these alternative, and likely complementary, interpretations.

Golgi tension data inconsistencies
The authors spread cells on FN and fixed cells over a time course, concluding that Golgi tension increases with cell spreading on FN. Again, the data appears to be different from t0 across the time course, but only for 2h and 4h. Oddly 3h is not significant. Again- micropatterning would be ideal for this. Plate cells on different areas and compare focal adhesion size, CARTS, Golgi tension and membrane area. Predicting that larger area would give you an increase in focal adhesions, CARTS number, and Golgi tension?

We agree that the temporal variability observed in the Golgi tension measurements (e.g., the lack of a significant difference at 3h, as also noted by reviewer #1) likely reflects biological and experimental variability inherent to cell spreading assays and FLIM measurement of Halo-Flipper lifetimes. We have now included an extra data point in that time course (please, see our related response to Reviewer #1; and **Fig. 3D***).*

As aforementioned, we agree that micropatterning approaches would provide a powerful and more controlled framework to systematically relate cell area, FA organization, CARTS biogenesis, and Golgi membrane tension. This is indeed an excellent suggestion that we plan to pursue in future work. Importantly, we note that our data do not support a strictly linear relationship between cell spreading area and CARTS number or Golgi tension, suggesting that additional regulatory layers contribute to this process. In the revised manuscript, we have clarified this point and tempered our interpretation accordingly.

They compare Golgi tension on soft and stiffer PAA to Glass, while 0.2 kPa reduces Golgi tension, it is only significant when compared to glass. Yet- the authors conclude "FLIM measurements showed significantly lower lifetimes on soft substrates relative to stiff ones,". Is the 30 kPa not considered stiff in this context? Cells can spread on the 30 kPa and form focal adhesions, but not on 2 kPa (Fig S1F). This data would then suggest that this does not alter the Golgi tension? (i.e. Fig 3D 2 kPa vs 30 kPa, Golgi tension is not significantly different between these two conditions p0.1680 means there is a 16.8% probability that any difference between the conditions is due to chance).

We thank the reviewer for highlighting this. We agree that our original wording overstated the differences in Golgi tension between substrate stiffness conditions. In particular, while we observe a significant reduction in Golgi membrane tension on soft substrates (2 kPa) compared to glass, the difference between intermediate (30 kPa) and soft or glass substrates is not statistically significant. In the revised manuscript, we have softened our conclusions accordingly and now state that Golgi tension is reduced under very soft conditions as compared to glass, while differences between intermediate and either glass or soft substrates are more subtle and not statistically significant in our measurements (similarly as before, if we compared the cell-by-cell variability measurements amongst the three conditions, we would end up with p-values $<10^{-3}$ for all comparisons: 2 kPa–30 kPa; 2 kPa–glass; 30 kPa–glass, using a non-matched ordinary one-way ANOVA test with Tukey's correction; N>90).

The role of microtubule stability
For me, this part of the project as presented is somewhat disconnected from the rest. They

abstractly rationalize that the microtubule sequestered Rho GEF, GEFH1, may be involved in this biology as GEFH1 activity is reported to activate PLCe>DAG>PKD. They chose to modify tubulin acetylation which controls GEFH1 association with microtubules. i.e microtubule acetylation promotes the release of GEF-H1 from microtubules into the cytoplasm. (I am not sure why they don't just directly test GEFH1? Or alphaTAT via shRNA). They concluded FN spreading increased microtubule acetylation in comparison to PLL but, they do not observe the increase in tubulin acetylation with a static 15% strain. (microtubule acetylation increases with compressive stress, not strain, so this is not surprising doi: 10.1038/s41563-023-01578-1). I am not sure why they included this as their earlier strain data was inconclusive too. (which conflicts with their statement on line 24, "Collectively, these results indicate that both physiological spreading forces as well as externally applied stretch enhance MT acetylation"???)
They increase tubulin acetylation with tubacin (HDAC6i) and see an increase in CARTs but no increase in cell area. Was niltubacin considered as a control? As tubacin can result in an increase in peripheral focal adhesions, could this be why CARTS increase in the Tubacin treatments?

We thank the reviewer for raising these points, which helped us clarify this part of our work. We agree that, in the original version of our manuscript, the connection between microtubule acetylation and the proposed signaling pathway was not sufficiently clear.

In the revised manuscript, we now explain more explicitly that our rationale is based on the reported roles of microtubule acetylation in regulating the release of GEF-H1 and subsequent RhoA-dependent signaling, which can in turn influence PLCε/DAG/PKD activity (Eisler et al., PMID: 30028295, 2018; Seetharaman et al., PMID: 34663953, 2022). We have also incorporated new data addressing the involvement of Rho-related signaling in our system (see response to Reviewer #1; and new **Fig. S3***), which we think strengthens this part of our study.*

We also agree that direct perturbation of GEF-H1 and/or αTAT1 would provide a more specific way to test this pathway. However, following the editorial guidance to prioritize other key experiments, we have not pursued these here and instead highlight them as important directions for future work.

Regarding the effects of mechanical stimulation, we thank the reviewer for pointing out the interesting work by Li et al. (PMID: 37386067, 2023), in which the authors have used cyclic stretch and observed no total changes in the length of acetylated MTs, but a significant change in their orientation (MTs being less oriented, i.e. with a smaller persistence length, in cyclically stretched condition). In line with this and our own data, we have revised the manuscript to correct our previous statement (previously line 24, page 11), and we now discuss this result more cautiously. It will be interesting in the future to investigate how stretch and compression cycles, as those performed in that paper, affect Golgi-derived carrier biogenesis.

Concerning the use of tubacin, we agree that HDAC6 inhibition may have pleiotropic effects, including possible effects on FA organization. We have therefore tempered our interpretation accordingly and now explicitly discuss the possibility that the observed increase in CARTS upon tubacin treatment may, at least in part, reflect indirect effects beyond tubulin acetylation. We agree that more specific perturbations will be needed to fully resolve this issue.

If CARTS export is blocked- can cells spread?

This was the most convincing data that Golgi trafficking is linked to spreading, but they fail to test the role of mechanics in this. They somewhat touch on this in the final figure showing when they inhibit PKD, they saw less CARTS with a decrease in area with a corresponding decrease in Golgi tension. They also specifically inhibit CARTS using a TGN46 KO, and saw a significant decrease in cell spreading. They should validate that there are less CARTS in this condition when cells spread. If integrin activation and FA formation require CARTS, is the converse true? Have the authors considered what happens if you block CARTS to FA size and integrin activation, given cells do not spread without CARTS

We thank the reviewer for their positive assessment of our data. We agree that this part of the study provides strong evidence linking Golgi-mediated trafficking to cell spreading.

Regarding the first point, steady-state reduction of CARTS formation in TGN46 KO cells has been shown previously in our earlier work (Lujan et al., PMID: 38466628, 2024), and we have now made this more explicit in the revised manuscript to clarify that impaired CARTS-dependent export is well-established.

*We also agree that the converse question raised by the reviewer (whether blocking CARTS formation affects FA organization and integrin activity) is highly relevant. Although a thorough analysis of FA dynamics will require extensive work, we have now started to address this experimentally. In particular, we examined integrin activation and FA organization in cells in which CARTS biogenesis was inhibited by PKD inhibition. Our new results (***Fig. S5C-E***) indicate that, although PKD inhibition reduces cell spreading, it does not cause a major change in the amount of active β1 integrin (in line with our previous results on active β1 integrin levels in gels of different stiffness). FA area is reduced after 4 h of PKD inhibition, in parallel with the decrease in overall cell adhesion area. These observations suggest that impaired Golgi export primarily limits the ability of cells to expand their adhesive area, rather than causing a strong change in integrin activation per se (see also our response to Rev. #1).*

*We have incorporated these new data into the revised manuscript (***Fig. S5C-E***) and discuss them in the context of the feedback relationship between Golgi export, cell spreading, and adhesion-dependent mechanoadaptation. At the same time, we agree with the reviewer that a more detailed analysis of FA dynamics and integrin trafficking under these conditions will be an important direction for future work.*

Minor

(1) Figure 1 is mislabeled:
(C), the adhesion surface area per cell (D), and the number of CARTS per unit adhesion area
They are labelled the other way around on the figure.
(2) Figure s4- please display the images using the same scale bars, particularly A DMSO 1h vs 4 h- this will make sure the reader understands that the size increase matches the increase in area shown in the graph).
(3) They say no FA are formed on PLL at 4 hours, but do not show the control FN cell to show FA on FN at 4h
(4) information on statistical methods used was lacking, more detail would be helpful.

We thank the reviewer for pointing these out, we have now amended them in the revised version. Regarding point (2), we appreciate the suggestion to standardize scale bars for easier visual comparison. We carefully considered this point; however, we chose to retain the current presentation, as we had decided to image the full cell to provide clearer information on cell morphology and adhesion geometry that we believe may be informative to the readers. To facilitate size comparison, we had clearly indicated cell dimensions using annotated arrows. We hope the reviewer finds this approach acceptable.

June 24, 2026

RE: JCB Manuscript #202510026R

Felix Campelo
Universitat Pompeu Fabra

Dear Dr. Campelo:

Thank you for submitting your revised manuscript entitled "Mechanical forces stimulate Golgi export". We would be happy to publish your paper in JCB pending final revisions necessary to meet our formatting guidelines (see details below) along with edits to address the final reviewer comments.

To avoid unnecessary delays in the acceptance and publication of your paper, please read the following information carefully.

A. MANUSCRIPT ORGANIZATION AND FORMATTING:

Full guidelines are available on our Instructions for Authors page, https://jcb.rupress.org/submission-guidelines#revised.
Submission of a paper that does not conform to JCB guidelines will delay the acceptance of your manuscript.

1) Text limits: Character count for Articles is < 40,000, not including spaces. Count includes abstract, introduction, results, discussion, and acknowledgments. Count does not include title page, figure legends, materials and methods, references, tables, or supplemental legends.

2) Figures limits: Articles may have up to 10 main text figures.

3) Figure formatting: Scale bars must be present on all microscopy images, including inset magnifications. Molecular weight or nucleic acid size markers must be included on all gel electrophoresis. Aspect ratios of images may not be altered.

4) Statistical analysis: Error bars on graphic representations of numerical data must be clearly described in the figure legend. The number of independent data points (n) represented in a graph must be indicated in the legend. Statistical methods should be explained in full in the materials and methods. For figures presenting pooled data the statistical measure should be defined in the figure legends. Please also be sure to indicate the statistical tests used in each of your experiments (either in the figure legend itself or in a separate methods section) as well as the parameters of the test (for example, if you ran a t-test, please indicate if it was one- or two-sided, etc.). Also, if you used parametric tests, please indicate if the data distribution was tested for normality (and if so, how). If not, you must state something to the effect that "Data distribution was assumed to be normal but this was not formally tested."

5) Abstract and title: The abstract should be no longer than 160 words and should communicate the significance of the paper for a general audience. The title should be less than 100 characters including spaces. Make the title concise but accessible to a general readership.

6) Materials and methods: Should be comprehensive and not simply reference a previous publication for details on how an experiment was performed. Please provide full descriptions in the text for readers who may not have access to referenced manuscripts.

7) All antibodies, cell lines, animals, and tools used in the manuscript should be described in full, including accession numbers for materials available in a public repository such as the Resource Identification Portal. Please be sure to provide the sequences for all of your primers/oligos and RNAi constructs in the materials and methods. You must also indicate in the methods the source, species, and catalog numbers (where appropriate) for all of your antibodies. Please also indicate the acquisition and quantification methods for immunoblotting/western blots.

8) Microscope image acquisition: The following information must be provided about the acquisition and processing of images:
a. Make and model of microscope
b. Type, magnification, and numerical aperture of the objective lenses
c. Temperature
d. Imaging medium
e. Fluorochromes
f. Camera make and model
g. Acquisition software
h. Any software used for image processing subsequent to data acquisition. Please include details and types of operations involved (e.g., type of deconvolution, 3D reconstitutions, surface or volume rendering, gamma adjustments, etc.).

9) References: There is no limit to the number of references cited in a manuscript. References should be cited parenthetically in the text by author and year of publication. Abbreviate the names of journals according to PubMed.

10) Supplemental materials: There are strict limits on the allowable amount of supplemental data. Articles may have up to 5 supplemental figures. Please also note that tables, like figures, should be provided as individual, editable files. A summary of all supplemental material should appear at the end of the Materials and methods section.

11) eTOC summary: A ~40-50-word summary that describes the context and significance of the findings for a general readership should be included on the title page. The statement should be written in the present tense and refer to the work in the third person.

12) Conflict of interest statement: JCB requires inclusion of a statement in the acknowledgements regarding competing financial interests. If no competing financial interests exist, please include the following statement: "The authors declare no competing financial interests." If competing interests are declared, please follow your statement of these competing interests with the following statement: "The authors declare no further competing financial interests."

13) ORCID IDs: ORCID IDs are unique identifiers allowing researchers to create a record of their various scholarly contributions in a single place. Please note that ORCID IDs are now *required* for all authors. At resubmission of your final files, please be sure to provide your ORCID ID and those of all co-authors.

14) A separate author contribution section following the Acknowledgments. All authors should be mentioned and designated by their full names. We encourage use of the CRediT nomenclature.

Please note that JCB now requires authors to submit Source Data used to generate figures containing gels and Western blots with all revised manuscripts. This Source Data consists of fully uncropped and unprocessed images for each gel/blot displayed in the main and supplemental figures. For assays performed using capillary electrophoresis and/or immunoassay-based detection, authors should instead provide the electropherogram graph(s) for each experiment, plotting fluorescence/chemiluminescence intensity vs. molecular weight/size. Please be sure to provide one Source Data file for each figure gels, blots, and/or capillary electrophoresis assays along with your revised manuscript files. File names for Source Data figures should be alphanumeric without any spaces or special characters (i.e., SourceDataF#, where F# refers to the associated main figure number or SourceDataFS# for those associated with Supplementary figures). For traditional gels and blots, the lanes of the gels/blots should be labeled as they are in the associated figure, the place where cropping was applied should be marked (with a box), and molecular weight/size standards should be labeled wherever possible. For capillary electrophoresis assays, each trace in the graph should be color-coded and labeled to indicate which protein, gene, or sample is being measured (please try to avoid red/green combinations to accommodate our color-blind readers).

Source Data files will be made available to reviewers during evaluation of revised manuscripts and, if your paper is eventually published in JCB, the files will be directly linked to specific figures in the published article.

Source Data Figures should be provided as individual PDF files (one file per figure). Authors should endeavor to retain a minimum resolution of 300 dpi or pixels per inch. Please review our instructions for export from Photoshop, Illustrator, and PowerPoint here: https://rupress.org/jcb/pages/submission-guidelines#revised

Journal of Cell Biology now requires a data availability statement for all research article submissions. These statements will be published in the article directly above the Acknowledgments. The statement should address all data underlying the research presented in the manuscript. Please visit the JCB instructions for authors for guidelines and examples of statements at (https://rupress.org/jcb/pages/editorial-policies#data-availability-statement).

B. FINAL FILES:

Please upload the following materials to our online submission system. These items are required prior to acceptance. If you have any questions, contact JCB's Managing Editor, Lindsey Hollander (lhollander@rockefeller.edu).

-- An editable version of the final text (.DOC or .DOCX) is needed for copyediting (no PDFs).

-- High-resolution figure and MP4 video files: See our detailed guidelines for preparing your production-ready images, https://jcb.rupress.org/fig-vid-guidelines.

-- Cover images: If you have any striking images related to this story, we would be happy to consider them for inclusion on the journal cover. Submitted images may also be chosen for highlighting on the journal table of contents or JCB homepage carousel. Images should be uploaded as TIFF or EPS files and must be at least 300 dpi resolution.

**It is JCB policy that if requested, original data images must be made available to the editors. Failure to provide original images

upon request will result in unavoidable delays in publication. Please ensure that you have access to all original data images prior to final submission.**

The license to publish form must be signed before your manuscript can be sent to production. A link to the license to publish form will be sent to the corresponding author only. Please take a moment to check your funder requirements before choosing the appropriate license.

Additionally, JCB encourages authors to submit a short video summary of their work. These videos are intended to convey the main messages of the study to a non-specialist, scientific audience. Think of them as an extended version of your abstract, or a short poster presentation. We encourage first authors to present the results to increase their visibility. The videos will be shared on social media to promote your work. For more detailed guidelines and tips on preparing your video, please visit https://rupress.org/jcb/pages/submission-guidelines#videoSummaries.

Thank you for your attention to these final processing requirements. Please revise and format the manuscript and upload materials within 14 days. If you need an extension for whatever reason, please let us know and we can work with you to determine a suitable revision period.

Please contact the journal office with any questions at cellbio@rockefeller.edu.

Thank you for this interesting contribution, we look forward to publishing your paper in Journal of Cell Biology.

Sincerely,

Johanna Ivaska
Monitoring Editor

Andrea L. Marat
Deputy Editor

Journal of Cell Biology

Reviewer #1 (Comments to the Authors (Required)):

Overall, the authors have addressed the points raised in my previous review in a satisfactory manner. The revised manuscript has been improved accordingly, and I do not have any further scientific comments. I only noticed a few minor grammatical/typographical issues (e.g. line 49: "Upon carrier fusion to the cell surface" should be "upon carrier fusion with the cell surface"; line 264: "is able to RhoA" - verb is missing). Other than that, I have no additional concerns.

Reviewer #3 (Comments to the Authors (Required)):

Overall I am pleased with the response to my suggestions and recommend the paper be accepted. To sufficiently address the query about the statistics/data display, I would like to see a supplementary table of all statistical analyses (mean, SD/SEM, for each biological replicate to match with the superplots method), with a clear definition of n. (i.e. n = cell). This would clarify the statistical approach.
my only other request would be to soften the wording around figure 2.
While a "trend" toward increased adhesion area is observed, the effect is not statistically significant. The statements that this "likely reflects" adhesion remodeling and the use of "consequently" appear to over-interpret the data. I gently suggest framing these explanations as speculative possibilities or limiting the discussion to conclusions directly supported by the statistical analysis. specifically, I would remove the word "consequently" as this implies causation which hasn't been directly demonstrated with the data as it stands.
congratulations to the authors, I look forward to seeing it in press.

2nd Revision - Authors' Response to Reviewers: July 1, 2026

Dear Andrea and Johanna,

Many thanks once again for handling our paper and for all the constructive comments, which helped improve the manuscript and will for sure inspire future work.

Following the final recommendations by Reviewers #1 and #3, we have made a few minor revisions in the text:

Reviewer #1 (Comments to the Authors (Required)):
Overall, the authors have addressed the points raised in my previous review in a satisfactory manner. The revised manuscript has been improved accordingly, and I do not have any further scientific comments. I only noticed a few minor grammatical/typographical issues (e.g. line 49: "Upon carrier fusion to the cell surface" should be "upon carrier fusion with the cell surface"; line 264: "is able to RhoA" - verb is missing). Other than that, I have no additional concerns.

We have modified the text accordingly (the verb missing was "activate" in line 264).

Reviewer #3 (Comments to the Authors (Required)):
Overall I am pleased with the response to my suggestions and recommend the paper be accepted. To sufficiently address the query about the statistics/data display, I would like to see a supplementary table of all statistical analyses (mean, SD/SEM, for each biological replicate to match with the superplots method), with a clear definition of n. (i.e. n = cell). This would clarify the statistical approach.

To make the statistical analyses and underlying quantitative data fully transparent, we have uploaded all GraphPad Prism files associated with the quantifications in the manuscript, including both main and supplementary figures, to a public Zenodo repository. These files contain the individual measurements, biological replicate structure, summary statistics, statistical tests, and exact P values used to generate the SuperPlots and associated analyses. The availability of these files is now stated in both the "Statistical analysis and data representation" section of the Methods and the new "Data availability" section.

my only other request would be to soften the wording around figure 2.
While a "trend" toward increased adhesion area is observed, the effect is not statistically significant. The statements that this "likely reflects" adhesion remodeling and the use of "consequently" appear to over-interpret the data. I gently suggest framing these explanations as speculative possibilities or limiting the discussion to conclusions directly supported by the statistical analysis. specifically, I would remove the word "consequently" as this implies causation which hasn't been directly demonstrated with the data as it stands.
congratulations to the authors, I look forward to seeing it in press.

We have modified that paragraph accordingly by changing "likely reflects" for "may reflect"; "Consequently" for "In addition"; and "indicate" for "suggest".

Sincerely,

Felix Campelo, on behalf of all authors
